# “Ya Luk Ka Tan Yoo”: An Ethnography of Filial Piety Culture, Medication Usage, and Health Perceptions of the Elderly in Rural Southern Thailand

**DOI:** 10.3390/ijerph191912134

**Published:** 2022-09-25

**Authors:** Luechai Sringernyuang, Tida Sottiyotin

**Affiliations:** 1 Faculty of Social Sciences and Humanities, Mahidol University, Nakhon-Pathom 73170, Thailand; 2 School of Pharmacy, Walailak University, Nakhon Si Thammarat 80161, Thailand

**Keywords:** elderly, ethnography, filial piety medicine, rural southern Thailand, the meaning of medication

## Abstract

Filial piety is a Buddhist virtue, and its meaning varies across cultures. In Thailand, filial piety refers to an appreciation of one’s indebtedness to others. Previous studies showed that filial piety is deeply grounded in longstanding culture values and related to the health of the elderly. Information from some literature revealed that medicinal products given to the elderly by their children, called “Ya-Luk-Ka-Tan-Yoo”, were apparent in the communities of rural southern Thailand. This study aims to explore in depth how “Ya-Luk-Ka-Tan-Yoo” is perceived, valued, and functions in southern Thailand’s socio-cultural contexts. Ethnography methodology is used, and a researcher was embedded in the field for six months, gathering data through participant observation and ethno-graphic interviews with fifty-two respondents. The findings reveal that filial piety medication is related to the local meanings of medicine, children, and gratitude. “Ya-Luk-Ka-Tan-Yoo,” in the eyes of both the elderly and their children, encompasses more than just health. Implicit herein are the concepts of a means of care and gratitude and a symbol of life. Filial piety medication is thus a carrier/medium of physical, financial, and emotional support. This research reveals how the ill health of the elderly is transformed to a commodity. Nonetheless, the negative impact of the efficacy of filial piety medication remains an issue of concern among professionals. The findings indicate that people are aware of the risks associated with self-medication. However, they insisted that their use was still necessary and justifiable.

## 1. Introduction

This paper aims to reveal insights and fill the gap of knowledge in how “ya-luk-ka-tan-yoo” or filial piety medicine is perceived, valued, and functioned in southern Thailand’s socio-cultural contexts.

Aging societies are an ongoing challenge for socioeconomic and healthcare systems internationally. Similar to many other countries, the traditional extended family structure in Thailand has gradually been replaced with smaller nuclear units [1]. Social changes that have occurred since the 1950s have affected the family institutions of Thai people in various ways. For example, the internal migration of family members for better education and economic opportunities (together with other factors like low fertility) have impacted their family structure and size. The number of skipped-generation families is increasing, especially in rural communities [1,2]. In these new contexts, family relationships are being transformed. Cultural values, such as love, respect, and gratitude, are now being maintained by material exchanges [3]. Furthermore, cultural diversity and family relations, especially in terms of filial piety, influence members’ health [4,5,6].

“Filial piety” is a Buddhist virtue, and its meaning varies across cultures. In the Confucian ethic, filial piety refers to a child’s support of their parent(s) [7]. A simplified definition is the appreciation of one’s indebtedness to others [8]. Filial piety affects elder wellbeing, especially in terms of their mental health [4,6]. The association of filial piety with loneliness, depressive symptoms, and stress has often been reported [4,6,9]. These studies clearly revealed that filial piety is deeply grounded in longstanding cultural values and is related to the health of the elderly. In addition, some literature pointed out the effects of this value on self-medication practices [3,10]. In Thailand, like elsewhere, self-medication practices have been commonly reported [11,12]. Such practices, especially among the elderly, are influenced by various factors. Information from the word-of-mouth of peoples’ friends, relatives, and other informal sources plays an influential role. Also reported is that medicinal products given to the elderly by their children, called “Ya-Luk-Ka-Tan-Yoo”, were apparent in the communities of rural southern Thailand [3,10]. Yet, details of how such practices are embedded in people’s everyday life are limited.

## 2. Materials and Methods

### 2.1. Study Design and Setting

This ethnographic study adopted constructivist ontology (the social phenomenon is socially constructed) and interpretive epistemology (realities are known through the interpretation of the meaning ascribed) to design methodologies and data analysis. The Consolidated Criteria for Reporting Qualitative Research (COREQ) and the Standards for Reporting Qualitative Research (SRQR) were adopted to assure the transparency of the qualitative methodologies and prepare the manuscript.

The researcher (TS) was embedded in the field, gathering data through participant observation and ethnographic interviews. Purposive sampling was used to select four communities in a district of Nakhon Si Thammarat Province (Thailand) based on socioeconomic criteria: the appearance of “Ya-Luk-Ka-Tan-Yoo” medication, characteristics of traditional southern culture in terms of livelihood and beliefs, availability of healthcare facilities similar to other areas in the region, and dynamics of changes and modernization.

### 2.2. Participants

A total of 52 participants (Table 1), including 32 elderly (aged 60 or over who had ever used “Ya-Luk-Ka-Tan-Yoo”), 15 family members of the elderly, and 5 other key informants were selected purposively.

Most of the elderly were female (65.63%), with primary educational level (91.67%), and were Buddhist. Four lived alone, with the rest living with either spouses or school-aged grandchildren. Each elderly had at least one health problem, including hypertension (91.67%), and they regularly received services from the hospital for hypertension (91.67%), hyperlipidemia (66.67%), and diabetes (41.67%).

Twelve elderly family members were children of the elderly, while the rest were relatives, nieces, or nephews. Most of these informants (twelve) lived away from their elderly, eight of which lived far away for more than five years. All informants kept track of their elderly relatives’ well-being and illnesses through phone calls. Most of them made daily calls and only visited their parents 1–2 times annually.

The other key informants consisted of three close neighbors, who were found being mentioned by the elderly informants as the ones who well know their health, daily life, and medication practices and two local health personnel who frequently did home visits to the elderly as part of their primary care service.

### 2.3. Data Collection and Trustworthiness

Informed consent both verbal and written was carried out with all participants, and the study protocol was approved by the appropriate ethics review board. The data collection was carried out between December 2020 and May 2021. One author (TS) was embedded in the research field around 12 h per week to gather data while maintaining a healthy balance of participation and observation [13]. Inter-subjectivity was used to foster acceptance, trust, and ultimately access to information through the researchers’ experiences with elderly family members and southern identity. Three main methods of data collection were used: participant observation and face-to-face interviews for 32 elderly, three family members who lived with the elderly, five key informants, and telephone interviews for 12 family members who lived away from their elderly. Content in observation and interview guidelines (Table 2) covered the definition of filial piety medication, use, and cultural beliefs and perceptions. After getting written or verbal consent, the interview began with general questions and then moved to more detail as pertinent or interesting issues arose. Permission for voice recording was also requested. The voice records were then transcribed verbatim. Field notes were taken to document the conversations’ atmosphere, situation, and scene. Additionally, the field notes also included a reflection on what had happened to identify any problems, obstacles, limitations, or prejudices that arose to improve our data collection methods to ensure that they would contain more in-depth information [14].

### 2.4. Data Analysis

The percentage of each type of medicine was calculated relying on the products found while visiting each case study. Each informant was asked to show all products used and available. All if the products were classified, counted, and then calculated. Content analysis was used for analyzing qualitative data. The author (TS) verbatim transcribed the audio data by herself. NVivo (Release 1.3)(QSR International, Victoria, Australia) was used to analyze the transcripts and field notes. Both authors read the transcripts several times to become acquainted with the data. Initial codes were established and then translated into themed maps. The authors outlined the final themes and sub-themes and then discussed matters to resolve any discrepancies. A conclusion was drawn from all indices, categorizations, and definitions by correlating their causes and outcomes. Finally, the first author translated all of the data from Thai to English.

### 2.5. Ethics Approval

The Human Research Ethics Committee of Walailak University (Thailand) approved this study under Certificate No. WUEC-20-325-01.

## 3. Findings

Living far away from home, leaving one’s aged parents, or having one’s grandparents experience their health deterioration alone is not ideal for most descendants, including those in the southern communities of Thailand. However, this is an unavoidable fact in the lives of many people in modern times. In their old age, most elderly expect psychological and financial support from their children. Previous studies have shown that receiving financial support and daily care from one’s children contributes to the physical and mental quality of life of the elderly [15,16]. In this context, we found that the cultural practice of filial piety medication links health commodities, elderly health, and traditional cultural values among Thai rural families.

“Ya-Luk-Ka-Tan-Yoo” translates as the medicine (ya) of a grateful (ka-tan-yoo) child (luk) and is a health product given to the elderly. Herein, it includes both healthcare and thankfulness.

Approximately 73% of the products of this treatment were found to be dietary supplements, whereas the remaining 17% were medicinal herbs and traditional medicines. Among these, collagen mixed products are most commonly perceived as good for one’s bones and joints, in addition to pain relief. The most popular dietary supplements are Colligi^®^ (collagen tripeptide powder), Nutrilite^®^ (plant protein), and multivitamin tablets. The average expense for these is 1028.52 Baht per month. The information sources for these medicines were primarily the TV and the Internet. Herein, parents would often request that their children find popular products that they had heard about from advertisements.

“Ya-Luk-Ka-Tan-Yoo,” in the eyes of both the elderly and their children, encompasses more than just health. Implicit herein are the concepts of (1) a means of care and gratitude and (2) a symbol of life (Table 3).

### 3.1. A Means of Care and Gratitude

#### 3.1.1. Aging Body and Care

Aging, illness, and care in the perceptions of the elderly and families are interrelated, and medicines are commonly meant to manage the health deterioration of the aged, as the elderly No. 2 said,

*“If you ask me why do older people need to take medicine? It is natural. When people become aged, their body breaks down, you need medicine, and then your life depends on it.”*

Health vulnerability often causes most elderly to regularly visit healthcare services. Traveling to health facilities is not always easy in rural areas, including within our study sites. There can be various difficulties like old age, health conditions, money, and transportation, etc., that cause the elderly to rely on others. In this context, medicines sent to them could ease this situation, practically and psychologically.

*“I could not go there (hospital) by myself; I am too old. An older man like me would not know what to do at the hospital. Everything is too difficult, so I wait for my son, wait for him to help…He (his son) takes these medicines in the meantime. My son sent these medicines for me to take while waiting for the doctor. He said that it would will not be so long until he takes me to see a doctor, he as he had promised.”*

*(Elderly No.11)*

#### 3.1.2. Thinking of

For families with distant children, medicines were used as a means of remembrance. From the perspective of the offspring, medication was not only due to the elderly health needs, it was also a medium that indicated that they were still worried, missed, and wanted to take care of their family members.

*“I think of my mother but I cannot go back to see her. I bought some medicines or dietary supplements instead of other products because (medicine or dietary supplement) is appropriate for the elderly’s health. I sent these medicines via Kerry (a private delivery company), it is very convenient. These medicines are like something to tell my mom that I miss her, I think of her, and I want to see her. I hope she would know my thinking of her.”*

*(Family member No.7)*

Several elderly people living along used these filial piety medications to symbolize the love and sentimentality of their children. The medications here function to maintain a sense of love-based bonding during times of separation. The case study of elderly No. 12 (64 years old) demonstrates this fact.

*“When our children grew up, and the economy was not good, they had to move away. As you can see, there are only two at home now. It’s lonely, but it is understandable. What makes me feel better is the fact that our children still think of us. [When asked, “How do you know that they still think of you?”] I can tell from both the medicines and foods that they bought and sent us here. It symbolized that they’re still thinking about us. Like these pills [lifts up a multivitamin bottle]. Our eldest child bought them for us. She told me that she was worried that she could not come to take care of us by herself, so she sent these to take care of us…This shows that they still think about us and are grateful to us. It makes me feel peaceful and that we are still family, just that they cannot be with us.”*

#### 3.1.3. Thankfulness

In a community where the family system and moral reciprocity are valued, medicines were often used as one of the means of returning the indebtedness.

*“It (Ya-Luk-Ka-Tan-Yoo) can be medicines or anything that helps maintain our parents, grandparents, or the elderly whom we respect and are grateful, healthy. Filial piety is what Buddhism has taught us we have to be grateful to other people and return their favors. Returning favors can be done in several ways, but I choose to buy the medicine because I think it is valuable. This is suitable for my parents. Also, I feel like it can compensate for the fact that I cannot take care of them by myself.”*

*(Family member no. 10).*

The story of elderly No. 2 (71 years old) demonstrates this. The old man had two daughters, one of which, the eldest, worked and lived in another province. His health was not good. Apart from high blood pressure and high cholesterol, he also suffered from dizziness. His oldest daughter often sends him various products, including dimenhydrinate and other popular multivitamin supplements.

*“My daughter told me that she was thankful for my love and care. She rarely comes home to take care of me. These (medications) are necessary. If you ask me whether I feel tired of taking these, I would say yes. Nevertheless, these are from my daughter, they make me feel cared for, and I am glad that she is a grateful child.”*

### 3.2. A Symbol of Life

#### 3.2.1. Social Status

The medicinal products received often have a deeper meaning then simply being used for health promotion. The use of branded, well-known, and expensive products causes elderly parents to feel proud of their social status, allowing them to show a sense of pride to their neighbors. Being given health products not only means that they have good children, but the brands also imply a good (economic) status. For example, elderly No. 5 (68 years old) said:

*“Whoever comes, they will see what I have. They will ask to take a look at this medicine [picks up a bottle of collagen], and they often joke with me that I am so trendy. This medicine my daughter bought and sent to me telling that it is good, because it’s an imported stuff. She bought them for me, so I am fashionable [laughs].”*

Family member No. 7, who gave some expensive medicines to her mom, expects that it ensures her mom will be accepted by surrounding people.

*“I try to buy good products even with a high price to be sure that I would get a good quality one so that when my mom tells or shows them to the others, she’ll not be embarrassed… When my mom saw an advert of this [picks up a product] on TV, she’d smile. Sometimes, she’d call people to see that she’s also using it [laughs]. It’s pretty expensive but I can afford it. It’s not a problem.”*

#### 3.2.2. Successful

One elderly informant informed a different perception of “Ya-Luk-Ka-Tan-Yoo”. Elderly No. 5 has one daughter, who lived far away for more than eight years. Her daughter has worked in a five-star hotel in Krabi province, made daily calls, and came back to her hometown only once a year. The daughter usually sends expensive medicines and health supplements to the elderly. The elderly did not perceive these medicines as a present but also perceived them as a symbol of a successful life.

*“I used to be poor and felt ashamed. I did not have anything to show off to others. Today, I am not. The people around me are jealous of me. My daughter received a high degree and earn a several-thousand (baht) salary, she sent some expensive stuff for me. I call this a success.”*

## 4. Discussion

Based on the Confucian perspective, filial piety encompasses both the material and emotional aspects of the parent–child relationship. The child is the parent’s supporter and their successor. Filial piety establishes both family norms and the social and moral foundations necessary for the maintenance of social order in a stable society [7]. The components of filial piety medication are similar to the Confucian ethic in which health products are used as materials. Filial piety is a virtue and primary obligation around showing respect, obedience, and care, all of which affect the health and way of life of Asian peoples. In particular, reciprocal filial piety affection is derived from long-term positive interactions with one’s parents in daily life and is rooted in the intimacy and quality of the parent–child relationship [7,17]. Filial piety medications act as a conduit for parents’ emotional and spiritual care. This tenet is consistent with several pharmaceutical-based anthropological studies reporting that medication is both a pharmacological substance and an ideological vehicle in people’s lives. For instance, medication embodies people’s perceptions of both illness and their responses to it, as well as one’s idea about the self, and can be used to indicate an individual’s taste and their social class [18,19,20,21].

Thailand’s rapid change in the aging population is ranked third in the world. It is expected that, by 2050, the aging population will increase to 20 million (35.8% of the population). This demographic change implies significant challenges for the country for care and support for older people [22]. The effects of this demographic transition on the quality of life and health of the elderly could be worsened by the dynamics of socio-economic contexts. Disparities of development that result in the rural-to-urban migration of the rural families’ offspring explain the lonely life of the elderly. Skipped-generation and small-sized family structures weaken life immunity, which makes the dependent members like the elderly more vulnerable to the negative consequences of social changes. Filial piety medication in this regard can be interpreted as the manifestation of the decay of rural family life. The cultural values of gratitude between the children and parents then are reinterpreted to fulfill the new needs.

Filial piety medication defines medication as a medium for users’ ideologies that correspond and relate to their sociocultural system’s context. For example, economic shifts and income declines have impacted elderly families in the rural south of Thailand, resulting in the emigration of working-aged people. However, people have still attempted to maintain their kinship systems and filial piety practices. This study found that filial piety medication exists in the daily lived experiences of the elderly in their physical, mental, and social dimensions. Medication has developed into a tangible manifestation of children’s and relatives’ reciprocity towards the elderly. It has evolved into a means of caring for the elderly’s declining physical health, promoting or elevating their social status, and fostering positive relationships among family members, all of which contribute to older family members’ mental wellbeing. Among rural families, their low educational level and socioeconomic status often affect their solid filial piety [7]. As such, medication for maintaining filial piety is critical herein. In emotional dependence, filial piety is inextricably linked to loneliness and mental health [4,6]. Filial piety medication is thus a carrier/medium of physical, financial, and emotional support.

Additionally, this research also reveals how the ill health of the elderly is transformed to a commodity. “Filial piety medication and dependence” is a phenomenon arising from the discourse around the aging of elderly people and their families. “Aging and disease” is a long-standing societal debate [23]. According to field data, the elderly and their families believe that their illnesses are typical. This is consistent with the findings of Venn and Arber, in which elderly informants described insomnia as a natural part of aging. It is not a health issue but a natural part of aging. Additionally, they viewed medicine as a necessary component of life that helped them cope with the aging process. Finally, they found that parents typically expect support from their children [24].

Nonetheless, the negative impact of filial piety medication remains an issue of concern among professionals. Our findings indicate that people are aware of the risks associated with self-medication. However, they insisted that their use was still necessary and justifiable. For example, people reported having no time to take their elderly relatives to the doctor, necessitating the purchase of medications to alleviate symptoms. Purchasing medication for one another was also an easy and natural way for community members to express gratitude. This is consistent with the research by Pedersen, Haslund-Thomsen, Curtis, and Grønkjær that described health as a constituent of both the individual and society. Herein, although the informants recognized that smoking and drinking were harmful behaviors, they insisted on continuing them because they were considered normal in society. The researchers brought up an intriguing discussion that, within different societies and cultures, people’s behaviors vary; hence, it is not possible to conclude that behaviors are seen as universally either good or bad, only whether they are acceptable in a given society [25].

The result of this study also show how the drug market is influential on individuals’ health and medication use. The pharmaceutical industry and manufacturers of health products use advertising as a meaningful tool with which to influence the public’s health perceptions, self-medication decision-making, and the development of a new culture among users. Some of these advertisements are even based on the stories of filial piety. Over 50% of the informants stated that they chose their filial piety medication based on advertisements that they had seen in various media sources. They primarily evaluated products based on their properties, safety, and recommendations from individuals whom the media claimed to be genuine users. Additionally, they evaluated their filial piety medications based on their peers’ acceptance. This finding is associated with analgesic use among participants with chronic pain in the study by Eaves, in which medication advertisements were found to serve as a communication tool that assists consumers in comprehending product information. Additionally, it generates demand for the product by promoting it as a way for consumers to develop a new identity or personality, such as by increasing their acceptance, uniqueness, and belonging. In modern society, many pharmaceutical companies are willing to spend money on these strategies when communicating directly to consumers, eventually leading to a new culture of medication use [26].

## 5. Conclusions

This research explored the cultural usage of filial piety medication among the elderly population in southern Thailand using a rural community in Nakhon Si Thammarat Province as a case study. The results reveal that filial piety medication is a reinterpretation of medication in the daily life of the elderly, which is relevant and intertwined with their local economic, social, and cultural systems. Therefore, to effectively implement policy on health or medicinal use, all relevant systems should be targeted simultaneously, while taking into account cultural values.

## 6. Study Limitation

A notable limitation of this research is the role of the researcher, who was an interviewer. Despite having advantages in having a background as a healthcare professional which should help provide a better understanding of community health systems, on the other hand, it may make informants feel like being counseled and having their behavior supervised, resulting in mistakenly more positive outcome. Additionally, due to the age difference between the researchers and the elderly informants, there might be some elderly jargon, behavior, or interview answers with hidden meanings that the researchers could not fully understand. However, we believe that the data collection by the main researcher, who was southern Thai, and the methodology that the researcher used, an ethnographic interview, can build trust from the informants, leading to reducing such effects in this study.

## 7. Recommendations

1. The quality of life of the elderly, especially in rural communities, should be used as an indicator for all development policies. Filial piety medication practices on the one hand could be seen as survival mechanisms of the family to cope with the negative effects of social changes. Yet, on the other hand, such practices could reflect the problems of elderly living conditions and the inaccessibility of primary care and health services.

2. The marketing practices of food supplements and other health products for the elderly should be more monitored or even controlled. It is also important that more emphasis should be placed on health literacy among the public.

3. Emphasis on self-sufficienct economic policy could be an alternative development paradigm that would help rural families in Thailand be more self-reliant. This might increase immunity in dealing with the unintended negative consequences of the social changes of the families.

4. The medical service system should prioritize the doctor–patient relationship to ensure that doctors understand their patient’s unique context and the fact that patients often feel comfortable disclosing their medications to their doctor. This would result in the increased provision of seamless care and the more effective prevention and monitoring of any adverse reactions associated with polypharmacy among the elderly.

## Figures and Tables

**Table 1:** Informants’ characteristics.

Characteristics	Informants (N = 52)		
	Elderly	Family Members	Other Key Informants
N	32	15	5
Age (years, average)	72.81	40.67	61.2
Gender			
Male	11	4	2
Female	21	11	3
Relationship with elderly			
Children	-	12	-
Relative, niece, or nephew	-	3	-
Close neighbor	-	-	3
Health personnel	-	-	2

**Table 2:** Observe and interview guide.

Objectives	Point to Observe	Main and Probing Questions
Perception of “filial piety medication”	1. Type and number of medicines	1. What is it/What does it mean?
	2. How and when elderly use OR how and when children offer	2. How to get it? How to use it?
	3. Feeling and reaction when elderly use OR feeling and reaction when children offer	3. What does it refer to?
		4. How is it important?
How “filial piety medication is valued in this context	1. Daily routine and life conditions	1. How do you use these medicines? Why do you do?
	2. Family relationship	2. How about your family? How long have you ever seen? How do you feel? How it related with your medicines?
	3. Social interaction	3. How about your neighbor/community? How do you feel? How it related with your medicines?
	4. External environment (public communication, public transportation, health system, etc.)	4. How about your community facilities? How do you feel? How it related with your medicines?

**Table 3:** Major themes and illustrative quotes identified through ethnographic interview.

Themes	Subthemes	Illustrative Quotes
A means of care and gratitude	Aging body and care	“When people age, their body breaks down, you need medicine, and then your life depends on it.” (Elderly No.2) “I could not go there (hospital) by myself; I am too old. An older man like me would not know what to do at the hospital. Everything is too difficult, so I wait for my son, wait for him to help…My son sent these medicines for me to take while waiting for the doctor. He said it wouldl not be so long to see a doctor, hepromised.” (Elderly No.11)
	Thinking of	“I can tell from both the medicines and foods they bought and sent us here. It symbolized that they’re still thinking about us…Our eldest child bought them for us. She told me that she’s worried that she could not come to take care of us by herself, so she sent these to take care of us.” (Elderly No.12) “I miss my children; I still miss them even now. They’ve sent us medicines and other stuff, so I know that they also miss us…It’s necessary that they have to be away but they haven’t died from us. They told that they didn’t know what to do to repay us, so they sent us these medicines” (Elderly No.25) “It gives me peace of mind that we’re still family. Our children still think of us and are grateful to us. They cannot be with us.” (Elderly No.28)
	Thankfulness	“I have medicines from both my doctor and my daughter. My daughter told me that she was thankful for my love and care. She rarely comes home to take care of me. These (medications) are necessary. If you ask me whether I feel tired of taking these, I would say yes. Nevertheless, these are from my daughter, they make me feel cared for, and I am glad that she is a grateful child.” (Elderly No.2) “It (Ya-Luk-Ka-Tan-Yoo) can be medicines or anything that helps maintain health for our parents, grandparents, or the elderly whom we respect and are grateful, healthy. Filial piety is what Buddhism has taught us-we have to be grateful to other people and return their favors. Returning favors can be done in several ways, but I choose to buy the medicine because I think it is valuable.” (Family member No. 10)
A symbol of life	Social status	“Whoever comes, they will see what I have. They will ask to take a look at this medicine (picks up a bottle of collagen), and they often joke with me that I am so trendy. This medicine my daughter bought and sent me telling that it is good, because it’s an imported stuff. She bought them for me, so I am fashionable (laughs).” (Elderly No.5) “I try to buy good products even with a high price to ensure that I would get a good quality one so that when my mom tells or shows them to the others she’ll not be embarrassed… When my mom saw an advert of this (picks up a product) on TV, she’d smile. Sometimes, she’d call people to see that she’s also using it (laughs). It’s pretty expensive but I can afford it. It’s not a problem.” (Family member No.7)
	Successful	“I used to be poor and felt ashamed. I did not have anything to show off to others. Today, I am not. The people around me are jealous of me. My daughters received a high degree and earn a several-thousand (baht) salary, she sent some expensive stuff for me. I call this a success.” (Elderly No.5)
